# Editorial: Neurotechnologies for Human Augmentation

**DOI:** 10.3389/fnins.2021.789868

**Published:** 2021-11-11

**Authors:** Davide Valeriani, Hasan Ayaz, Nataliya Kosmyna, Riccardo Poli, Pattie Maes

**Affiliations:** ^1^Neurable Inc., Boston, MA, United States; ^2^School of Biomedical Engineering, Science and Health Systems, Drexel University, Philadelphia, PA, United States; ^3^Media Lab, Massachusetts Institute of Technology, Cambridge, MA, United States; ^4^School of Computer Science and Electronic Engineering, University of Essex, Colchester, United Kingdom

**Keywords:** brain-computer interface, neuroergonomics, human augmentation, neural decoding, brain stimulation, neurotechnology

Neurotechnologies combine neuroscience and engineering to create tools for studying, repairing, and enhancing brain function. Traditionally, researchers have used neurotechnologies, such as Brain-Computer Interfaces (BCIs), as assistive devices, for example to allow locked-in patients to communicate. In the last few decades, non-invasive brain imaging devices, such as electroencephalography (EEG) and functional near-infrared spectroscopy (fNIRS), have become more portable and inexpensive, paving the way to innovative applications of neurotechnologies (Ayaz and Dehais, 2018). Recent trends in neuroergonomics and neural engineering have used neurotechnologies to enhance various human capabilities, including (but not limited to) communication, emotion, perception, memory, attention, engagement, situation awareness, problem-solving, and decision making (Cinel et al., 2019; Kosmyna and Maes, 2019).

This Research Topic provides a collection of 12 contributions on recent advances in the development of non-invasive BCIs for human augmentation, with a particular emphasis on brain stimulation and neural decoding.

To introduce the topic of human augmentation, Dehais and colleagues propose a two-dimensional framework that incorporates arousal and task engagement to characterize different variables typically used in human augmentation, such as mental workload and human performance (Dehais et al., 2020). Specifically, poor task engagement leads to mind wandering or effort withdrawal depending on arousal level, while a too high arousal could lead to perseveration or in attentional blindness and deafness. Neurotechnologies could, therefore, be used to guide the brain to an optimal position in the arousal-engagement space to maximize performance, a position characterized by medium levels of arousal and high task engagement, which could be achieved, for example, by using brain stimulation or neurofeedback.

A few studies in this Research Topic investigated the use of non-invasive brain stimulation to augment human performance: a very popular topic in the area of neurotechnologies (Kadosh, 2014; Santarnecchi et al., 2015). Pilly and colleagues propose a novel paradigm based on virtual reality to use transcranial electrical stimulation (tES) to extend long-term metamemory (Pilly et al.). By applying periodic brief pulses while participants were asleep, they improved memory recall of one-shot viewing of naturalistic episodes over 48 h by 10–20%. Patel and colleagues performed a systematic meta-analysis to review the use of transcranial direct-current stimulation (tDCS) for improving motor performance in upper limbs (Patel et al.). Brain stimulation significantly reduces reaction time, task execution time, and increases force and accuracy in elbow flexion tasks. Wang and colleagues reported that combining brain stimulation with physical training increases motor-evoked potential (MEP) amplitude and muscle strength, and decreases the dynamic posture stability index, reaction time, and error rate in motor learning tasks (Wang et al.). Similarly, Hollis and colleagues explored the use of transcranial static magnetic field stimulation (tSMS) to facilitate motor learning in healthy children. They found that tSMS did not increase MEP amplitude in children (as found by Wang and colleagues in adults), suggesting that age is a critical factor for the effectiveness of brain stimulation. Yet, they found tSMS inhibited early motor learning and facilitated later stage motor learning in the non-dominant hand, which motivated future investigations of tSMS as a potential non-invasive therapy for children with cerebral palsy (Hollis et al.).

Another set of studies focused on using non-invasive neuroimaging to decode specific mental states, which could provide further insights into brain activity. Asgher and colleagues used fNIRS and deep learning to estimate four different levels of mental workload in human participants (Asgher et al.). While traditional machine learning algorithms reached accuracies below 70%, convolutional neural networks with long short-term memory layers achieved significantly better performance of almost 90% accuracy across the four classes. These results exemplify the potential of deep learning in neural decoding for human augmentation. In another contribution, Klaproth and colleagues used passive BCIs to track perception and auditory processing of pilots during operations (Klaproth et al.). In particular, they found that a passive BCI could use EEG to distinguish between task-relevant and irrelevant alerts received by the pilot, hence improving situation awareness. This work demonstrates how passive BCIs could work as monitoring devices in a practical scenario without disrupting the main task.

Another neural decoding problem with direct applications in BCI research is mental imagery. Wairagkar and colleagues showed that temporal patterns extracted from EEG activity are sufficient to achieve single-trial classification of five different mental imagery tasks (Wairagkar et al.). These patterns can, therefore, be used as control signals of non-invasive BCIs, which could translate them into commands for external devices. Also in the area of neural decoding, Li and colleagues have shown the possibility of using advanced machine learning and signal processing techniques to decode emotions from EEG signals (Li et al.). In this domain, other work has tackled this challenge using more invasive recordings (Sani et al., 2018). Yet, to enable broadly-applicable human augmentation, similar results have to be achieved with non-invasive devices, such as the EEG used by Li and colleagues, which pose fewer ethical and socio-economic barriers than invasive devices.

Another study tackles the exciting area of speech decoding, which aims at translating brain activity into meaningful speech. This problem has been extensively tackled using invasive recordings, such as electrocorticography (Herff et al., 2015; Herff and Schultz, 2016; Angrick et al., 2019; Anumanchipalli et al., 2019). Here, Dash and colleagues demonstrated that this is possible even with non-invasive and, therefore, more practical neural recording devices, such as MEG (Dash et al.).

The transition to non-invasive, real-world BCIs for human augmentation would require strategies to enhance the limited signal quality recorded from the brain. As such, multimodal BCIs depending on a combination of physiological signals will be increasingly important. In that domain, Stuldreher and colleagues determined the synchrony between EEG, heart rate, and electrodermal activity while participants were engaged in an auditory task (Stuldreher et al.). They found that each modality works well in certain scenarios, and that merging all modalities into a unique metric seems most robust across a broad range of applications.

Finally, the development of new non-invasive neurotechnologies presents many opportunities for clinical and field applications as well as multifaceted new challenges (Dehais et al., 2020). In a review paper of this Research Topic, Gaudry and colleagues delve into the neuroethical issues that we might face in the upcoming decades as neurotechnologies transition from research to practice, and even home and office settings (Gaudry et al.).

We hope this Research Topic provides the reader with updates on recent advances in the area of non-invasive neurotechnologies for human augmentation. We would like to thank all authors who contributed, the reviewers who provided invaluable and timely feedback to the authors, and Dr. Eleonora Adami for designing the cover picture of this Research Topic.

## Author Contributions

DV wrote the first draft of the manuscript. All authors contributed to manuscript revision, read, and approved the submitted version.

## Conflict of Interest

DV is an employee of Neurable Inc. The remaining authors declare that the research was conducted in the absence of any commercial or financial relationships that could be construed as a potential conflict of interest.

## Publisher's Note

All claims expressed in this article are solely those of the authors and do not necessarily represent those of their affiliated organizations, or those of the publisher, the editors and the reviewers. Any product that may be evaluated in this article, or claim that may be made by its manufacturer, is not guaranteed or endorsed by the publisher.
